# An atypical presentation of cardiac tamponade and periorbital swelling in a patient with eosinophilic granulomatosis with polyangiitis: a case report

**DOI:** 10.1186/s13256-017-1434-9

**Published:** 2017-09-24

**Authors:** Alexandra C. Keefe, Joseph C. Hymas, Lyska L. Emerson, John J. Ryan

**Affiliations:** 1 Department of Medicine, University of Utah School of Medicine, Salt Lake City, UT USA; 2 General Cardiology, St. Luke’s Hospital, Twin Falls, ID USA; 3 Department of Pathology, University of Utah School of Medicine and ARUP Laboratories, Salt Lake City, UT USA; 4 Division of Cardiovascular Medicine, University of Utah Health Science Center, 30 North 1900 East, Room 4A100, Salt Lake City, UT 84132 USA

**Keywords:** Churg-Strauss syndrome, Cardiac tamponade, Vasculitis, Periorbital swelling, Effusion

## Abstract

**Background:**

Eosinophilic granulomatosis with polyangiitis is a rare, necrotizing systemic vasculitis associated with asthma and hypereosinophilia. Its cause and pathophysiology are still being elucidated.

**Case presentation:**

We report a case of eosinophilic granulomatosis with polyangiitis in a 50-year-old Caucasian woman who presented with chest pain, dyspnea at rest, fever, and periorbital swelling. She was found to have significant hypereosinophilia and cardiac tamponade physiology. A biopsy confirmed extensive infiltration of both lungs and pericardium by eosinophils. She did not have any anti-neutrophil cytoplasmic antibodies.

**Conclusions:**

Eosinophilic granulomatosis with polyangiitis diagnosis does not require the presence of anti-neutrophil cytoplasmic antibodies. Anti-neutrophil cytoplasmic antibody-positive and anti-neutrophil cytoplasmic antibody-negative eosinophilic granulomatosis with polyangiitis may present with different clinical phenotypes, perhaps suggesting two distinct disease etiologies and distinct pathophysiology.

## Background

Eosinophilic granulomatosis with polyangiitis (EGPA), formerly known as Churg-Strauss syndrome, is a rare, necrotizing systemic small-vessel vasculitis associated with asthma and hypereosinophilia [1]. In 1990, the American College of Rheumatology (ACR) established six criteria to aid in the clinical diagnosis of Churg-Strauss syndrome based on extensive analysis of different patient presentations. These criteria include a diagnosis of: (1) asthma, (2) eosinophilia > 10%, (3) neuropathy (mono or poly), (4) non-fixed pulmonary infiltrates, (5) paranasal sinus abnormality, and (6) extravascular eosinophils [2]. The ACR determined that having at least four of the six criteria yields a sensitivity of 85% and a specificity of 99.7% for Churg-Strauss syndrome [2]. These criteria are still used for the diagnosis of EGPA today.

We present an unusual case of a patient who met four of the six criteria for the diagnosis of EGPA, but also had several clinical phenotypes that have rarely been reported in the literature. We discuss the role that her anti-neutrophil cytoplasmic antibodies (ANCA) status may play in the pathophysiology of her disease.

## Case presentation

**Fig. 1:**
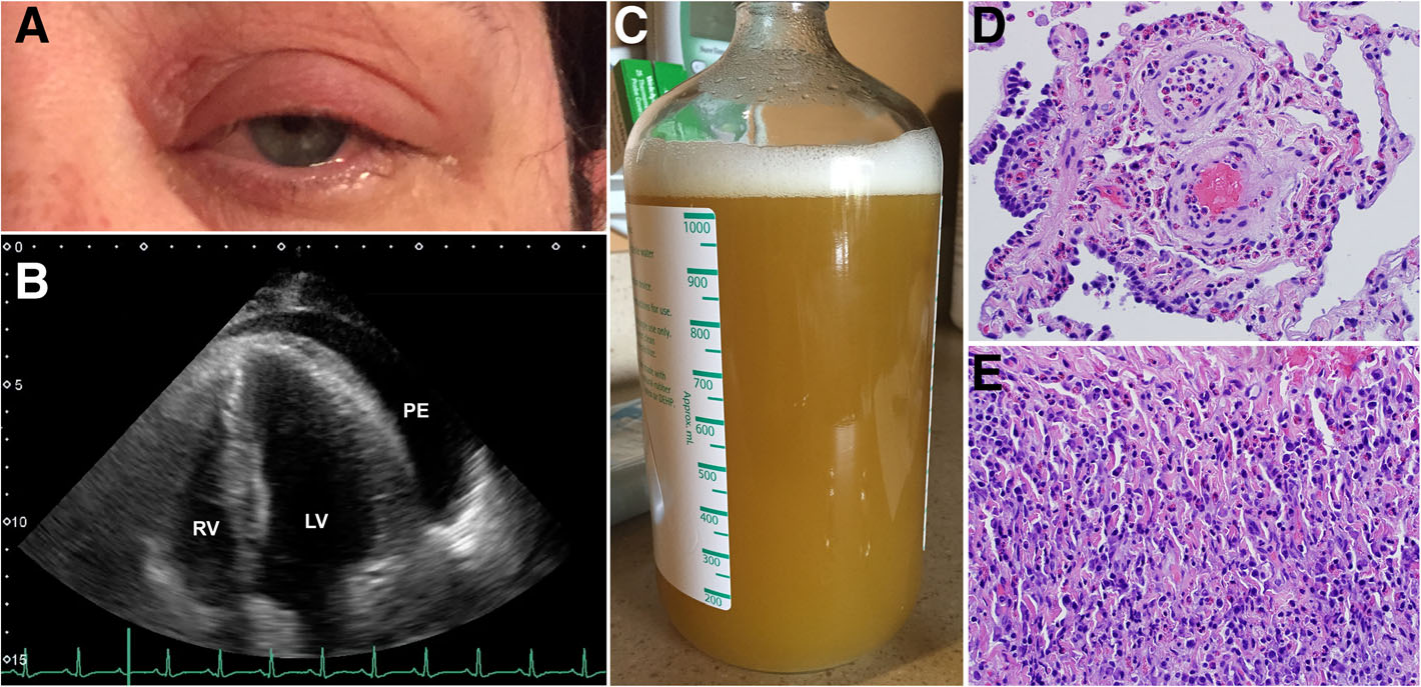
Unusual clinical presentations of anti-neutrophil cytoplasmic antibody-negative eosinophilic granulomatosis with polyangiitis. **a** Our patient showing left periorbital swelling, possibly representing an orbital inflammatory pseudotumor. **b** The apical chamber view of a transthoracic echocardiogram shows a significant pericardial effusion. **c** Fluid drained from the pleural effusion contained 77% eosinophils. **d** Biopsied lung tissue shows marked infiltration by eosinophils, with eosinophils infiltrating the walls of small vessels, the submucosa of an adjacent small airway, and the interstitium. **e** Pericardial tissue shows significant infiltration by both eosinophils and plasma cells. **d** and **e** Hematoxylin and eosin, × 400. *LV* left ventricle, *PE* pericardial effusion, *RV* right ventricle

A 50-year-old Caucasian woman presented with chest pain, dyspnea at rest, fever, chills, night sweats, and left periorbital swelling (Fig. 1a). Her past medical history was significant for adult-onset asthma, hay fever, sinusitis, and recurrent nasal polyps. An initial transthoracic echocardiogram revealed a significant pericardial effusion (PE) with tamponade physiology (Fig. 1b). Pericardiocentesis was performed. She recovered well and was discharged. Ten days later, she presented with a syncopal episode. An echocardiogram showed a recurrent PE and a large pleural effusion. Thoracentesis was performed, and one liter of brown frothy fluid was removed, containing 77% eosinophils (Fig. 1c). A complete blood count revealed 45% eosinophilia. Antibodies to *Trypanosoma cruzi*, *Strongyloides*, and ANCA were not present. Cytology, flow cytometry, and bone marrow aspirate showed no evidence of a hematopoietic malignancy. She underwent video-assisted thoracoscopic surgery for drainage of pericardial and pleural effusions. Subsequent biopsy of the lung and pericardium showed marked tissue infiltration by eosinophils (Fig. 1d, e). She was diagnosed as having EGPA, formerly known as Churg-Strauss syndrome. She was started on high-dose prednisone and her eosinophilia and clinical symptoms resolved. Cyclophosphamide was added before discharge to help prevent symptom recurrence.

## Discussion

Our patient presented with asthma, paranasal sinus abnormalities, hypereosinophilia, and extravascular eosinophils, and therefore met four of six criteria needed for the diagnosis of Churg-Strauss syndrome [2]. Of interest, she did not present with any neuropathy or pulmonary infiltrates, which are the two other criteria used to classify EGPA and are some of the more common manifestations of this disease [2, 3]. Instead, she exhibited left periorbital swelling, probably representing a so-called orbital inflammatory pseudotumor formed by a localized mixed inflammatory cell infiltrate which, in our case, would be expected to also include numerous tissue eosinophils [4]. Very few cases of orbital inflammatory pseudotumors associated with EGPA have ever been described and may only present in less than 1% of all patients with the disease [4–6]. In addition, her cardiac tamponade was another unusual clinical presentation, reported in only ten other cases of EGPA [3, 7, 8]. Three of those studies were also able to confirm eosinophilic infiltration in the pericardium with biopsy, as we have demonstrated (Fig. 1e) [8]. Since the cause of EGPA is unknown and is difficult to study due to low disease prevalence (less than 1.4 per 100,000 adults), unusual case presentations like ours are an important step in helping to elucidate how different clinical phenotypes correlate with disease pathophysiology [3].

One hypothesis for why our patient had such an uncommon clinical presentation of her disease was because she lacked ANCA. Of interest, while EGPA is considered an ANCA-associated systemic vasculitis, only ~ 40 to 60% of patients with EGPA are ANCA-positive, as compared to up to 95% of patients with other small vessel vasculitides [3, 7, 9]. This is why the presence of ANCA is not a requirement for EGPA diagnosis. One study which stratified the clinical symptoms of patients with EGPA according to their ANCA status showed that ANCA-negative patients with EGPA are more likely to have fever and cardiac anomalies (more likely pericarditis and cardiomyopathy, less likely tamponade), whereas ANCA-positive patients more often had peripheral neuropathy, vasculitis, and renal involvement [7]. These findings have led several groups to hypothesize that the pathophysiology of ANCA-positive and ANCA-negative EGPA may be different. To generalize, it has been observed that ANCA-positive EGPA may have a more “vasculitic” phenotype, characterized by small vessel vasculitis, whereas ANCA-negative EGPA could be considered a more “eosinophilic” phenotype in which eosinophilic infiltration causes significant organ damage [7, 10]. This hypothesis will need further modeling and testing, but our unusual case would certainly lend support to this theory.

## Conclusions

This case provides an example of an unusual clinical presentation of ANCA-negative EGPA with significant hypereosinophilia leading to multisystem organ damage and unusual disease manifestations including cardiac tamponade and periorbital swelling. Further studies and modeling will be needed to determine whether ANCA-negative EGPA and ANCA-positive EGPA truly define two distinct diseases with different pathophysiology.

## Abbreviations

ACR: American College of Rheumatology
ANCA: Anti-neutrophil cytoplasmic antibodies
EGPA: Eosinophilic granulomatosis with polyangiitis
PE: Pericardial effusion
